# Fatty fish and fish omega-3 fatty acid intakes decrease the breast cancer risk: a case-control study

**DOI:** 10.1186/1471-2407-9-216

**Published:** 2009-06-30

**Authors:** Jeongseon Kim, Sun-Young Lim, Aesun Shin, Mi-Kyung Sung, Jungsil Ro, Han-Sung Kang, Keun Seok Lee, Seok-Won Kim, Eun-Sook Lee

**Affiliations:** 1 Cancer Epidemiology Branch, Division of Cancer Epidemiology and Management, Research Institute, National Cancer Center, Gyeonggi, South Korea; 2 Department of Food and Nutrition, Sookmyung University, Seoul, South Korea; 3 Center for Breast Cancer, National Cancer Center Hospital, National Cancer Center, Gyeonggi, South Korea; 4 Department of Surgery, College of Medicine, Korea University, Seoul, South Korea

## Abstract

**Background:**

Although it is believed that fish ω-3 fatty acids may decrease breast cancer risk, epidemiological evidence has been inconclusive. This study examined the association between fish and fish ω-3 fatty acids intake with the risk of breast cancer in a case-control study of Korean women.

**Methods:**

We recruited 358 incident breast cancer patients and 360 controls with no history of malignant neoplasm from the National Cancer Center Hospital between July 2007 and April 2008. The study participants were given a 103-item food intake frequency questionnaire to determine their dietary consumption of fish (fatty and lean fish) and ω-3 fatty acids derived from fish (eicosapentaenoic acid (EPA), and docosahexaenoic acid (DHA)).

**Results:**

Using a multivariate logistic regression model, high intake of fatty fish was associated with a reduced risk for breast cancer in both pre- and postmenopausal women (OR [95% CI] for highest vs. lowest intake quartiles, *p *for trend: 0.19 [0.08 to 0.45], *p *< 0.001 for premenopausal women, 0.27 [0.11 to 0.66], *p *= 0.005 for postmenopausal women). Similarly, reductions in breast cancer risk were observed among postmenopausal subjects who consumed more than 0.101 g of EPA (OR [95% CI]: 0.38 [0.15 to 0.96]) and 0.213 g of DHA (OR [95% CI]: 0.32 [0.13 to 0.82]) from fish per day compared to the reference group who consumed less than 0.014 g of EPA and 0.037 g of DHA per day. Among premenopausal women, there was a significant reduction in breast cancer risk for the highest intake quartiles of ω-3 fatty acids (ORs [95% CI]: 0.46 [0.22 to 0.96]), compared to the reference group who consumed the lowest quartile of intake.

**Conclusion:**

These results suggest that high consumption of fatty fish is associated with a reduced risk for breast cancer, and that the intake of ω-3 fatty acids from fish is inversely associated with postmenopausal breast cancer risk.

## Background

Breast cancer is one of the most prevalent cancers in the world including South Korea [1,2]. The second report by the World Cancer Research Fund and the American Institute for Cancer Research indicates that food and nutrition may affect the status of hormones that can modify breast cancer risk [3]. Among the dietary factors, there has been mixed evidence regarding the impact of fish and ω-3 fatty acid intake on breast cancer risk. Animal studies have demonstrated that a diet containing α-linolenic acid-rich linseed oil is very effective in arresting mammary tumor progression [4], and fish oil or a diet containing EPA or DHA can suppress tumor growth and inhibit metastases formation [5,6]. Ecological studies have suggested inverse relations between fish and fish ω-3 fatty acid intake and breast cancer risk [7,8]. However, results from case-control or cohort studies varies depending on the study design [9] and study populations [10-13]. Most studies on fish consumption and breast cancer are limited by their lack of distinction between fatty (blue) and lean (white) fish. The association between fatty and lean fish consumption and breast cancer risk was examined in a large nationwide case-control study in Sweden [14], though a weak, inverse association of dietary fish intake and breast cancer was detected (not significant), no clear difference was observed based on the type of fish. In contrast, the Norwegian Women and Cancer Study [15] found no association between salmon consumption and breast cancer risk. A recent large multi-center European Prospective Investigation into Cancer and Nutrition (EPIC) study suggested that there was no association between total, lean, or fatty fish intake with breast cancer risk. The results were not affected by menopausal status, although there was a positive association in the highest quintile for fatty fish with no statistically significant test for trend [10]. Stipp *et al*. [16] found a positive association between total fish intake and breast cancer risk, but the type of fish or preparation method played no significant role. The authors suggested that other factors associated with fish intake, apart from ω-3 fatty acids, might be responsible for this association.

This study investigated the association between fish intake and the incidence of breast cancer in Korean women. It was designed to investigate the possible effects of ω-3 fatty acid consumption using a case-control breast cancer-study design. We evaluated per capita energy and nutrient intake with particular emphasis on the intake of total fish (categorized into fatty and lean fish) and fish ω-3 fatty acids (total ω-3 fatty acids, eicosapentaenoic acid (EPA), and docosahexaenoic acid (DHA)).

## Methods

### Study subjects

Eligible breast cancer patients were enrolled at the Center for Breast Cancer, National Cancer Center Hospital, Korea between July 2007 and September 2008. Among 424 incident breast cancer patients aged 25 to 77 years old admitted for surgery, 398 patients agreed to participate in the study. After patients with a previous history of cancer or an inability to participate in the interview were excluded, 362 patients were eligible for enrollment. During the same period, the control group was enrolled at the Center for Early Detection and Prevention at the same hospital. Visitors to the Center for Early Detection and Prevention received health check-ups, including screening for five major cancers (stomach, colorectum, liver, breast, and uterine cervix) based on their eligibility for the National Cancer Screening Program [17]. Among 2,503 women who were contacted by the interviewers, 1,489 agreed to participate in the study. After excluding women with a history of malignant neoplasm or benign breast diseases and those that failed to complete the FFQ, 617 were eligible for inclusion. Participants who reported an implausible daily energy intake (≤ 600 kcal or ≥ 3500 kcal) were excluded (5 cases and 2 controls), and the controls were frequency-matched to cases using a 5-year age distribution. Final analysis was done for 358 cases and 360 controls. Study protocols and consent forms were approved by the institutional review board of the National Cancer Center Hospital (IRB protocol number NCCNCS 07-083), and all subjects provided informed consent for study participation.

### Data collection

A trained dietitian collected information on participant demographics and lifestyle factors (e.g., smoking habits, alcohol intake, and physical activity), using a structured questionnaire. Reproductive information was also collected (e.g., age at menarche, menopause status, age at menopause, menopausal status, postmenopausal hormone use, and parity). Smoking history was categorized as none, past, or current. A food frequency questionnaire (FFQ) was developed and validated to determine regular dietary intake. The reliability and validity of the FFQ have been previously reported [18]. Subjects were presented with a list of 103 food items and queried on the average frequency and the typical portion sizes of the specific foods eaten during the previous year. The average daily nutrient intake for each subject was measured by adding the intake amount and associated nutrient content per 100 g for each of the 103 foods. This value was converted to a daily nutrient intake using the scales for consumption frequency (i.e., never or rarely, once a month, two or three times a month, once or twice a week, three or four times a week, five or six times a week, once a day, twice a day, and three times a day) and portion size (i.e., small, medium, and large) included in the food frequency questionnaire. Eight fish items, covering 6 fatty fishes and 17 lean fishes, were included in the FFQ. The eight items were raw fish, blue (fatty) fish, hair tail, eel, yellow croaker/sea bream/flat fish, Alaskan pollack/Alaskan pollack (frozen)/Alaskan pollack (dried), anchovy/anchovy (marinated), and tuna (canned). We classified the types of fishes consumed (fatty and lean fish) to calculate the estimated amount of fatty acid consumption (EPA and DHA) and determine the effect of each fatty acid on breast cancer risk. The validity of the FFQ used in the current study has been tested using the 3-day dietary record as a gold standard in a total of 202 persons. The de-attenuation correlation coefficients, percent agreements of the same plus adjacent quartile categories, and percent gross misclassification were 0.491, 75.2% and 8.3% for total *ε*-3 fatty acids, respectively, 0.482, 70.6%, and 10.1% for EPA, respectively, and 0.549, 74.3%, and 5.5% for DHA, respectively.

### Statistical analysis

Alcohol consumption was categorized as either have or have not consumed alcohol. Physical activity was measured using the short form of the International Physical Activity Questionnaire (IPAQ) and summarized into metabolic equivalent (MET) units (minutes/week). Odds ratios (ORs) and 95% confidence intervals (CIs) were calculated, and the significance level was set at 5% for all statistical tests. The Chi-square and t-tests were used to compare characteristics between cases and controls. The consumed amounts of energy, fishes, and ω-3 fatty acids of cases and controls were compared using the t-test. Intake quartiles for fish and ω-3 fatty acids were categorized based on the intake values of control group. The SAS 9.1 (SAS Institute Inc., Cary, NC) LOGISTIC procedure was utilized to calculate odds ratios and their confidence intervals for fish and ω-3 fatty acids intake quartiles on breast cancer risk. Data were stratified by menopausal status. Multivariate models were adjusted for age, body mass index (BMI), family history of breast cancer, dietary supplement use, education level, occupation, alcohol consumption, smoking status, physical activity, age at menarche, parity, total energy intake, postmenopausal hormone use, menopausal status, and age at menopause. Especially, energy-adjusted nutrient intakes were computed as the residuals from the regression model with total caloric intake as the independent variable and absolute nutrient intake as the dependent variable [19]. To test for linear trends across fish and ω-3 fatty acids quartiles, the median intake of each quartile category was used as a continuous variable to test for trends.

## Results

The general characteristics of the study subjects are presented in Table 1. The mean ages of cases and controls were 48.3 and 47.9 years of ages, respectively, which were not statistically different. There were significant differences between the cases and controls for BMI (p = 0.003), dietary supplement use (p = 0.001), education (p < 0.001), occupation (p = 0.012), age at menarche (p < 0.001), and postmenopausal hormone use (p < 0.001). The amounts of fish and fish ω-3 fatty acids consumed by cases and controls are presented in Table 2. In general, the cases had significantly lower total fish (p = 0.012) and fatty fish intake (p < 0.001), but a higher energy intake (p = 0.032). With regard to menopause status, premenopausal breast cancer patients had a lower intake of fatty fish than controls (p < 0.001). Postmenopausal breast cancer patients consumed lower amounts of total fish (p = 0.022), fatty fish (p < 0.001), ω-3 fatty acids (p < 0.001), EPA (p < 0.001), and DHA (p < 0.001), but had a higher energy intake than controls (p = 0.039).

**Table 1:** General Characteristics of Study Subjects

Variables	Control (n = 360)	Case (n = 358)	*P*
**Age (years)^a^**	47.9 ± 8.7	48.3 ± 8.6	0.633
**Body mass index (kg/m^2^)**			
< 18.5	8(2.2)	16(4.5)	0.003
18.5- < 23	150(42.4)	153(42.7)	
23- < 25	121(34.2)	85(23.7)	
≥ 25	75(21.2)	104(29.1)	
**Family history (yes)**	12(3.6)	17(4.8)	0.429
**Supplement use (yes)**	167(67.1)	194(54.3)	0.001
**Marital status**			
Married	288(83.0)	289(80.7)	0.588
Single	14(4.0)	20(5.6)	
Divorced, Widowed, Other	45(13.0)	49(13.7)	
**Education**			
≤ Elementary school	21(6.1)	59(16.5)	< 0.001
Middle school	17(4.9)	44(12.3)	
High school	176(51.2)	174(48.6)	
≥ College	130(37.8)	81(22.6)	
**Occupation**			
Housewife	212(61.1)	217(60.6)	0.012
Profession, Office worker	75(21.6)	54(15.1)	
Sales, Service	43(12.4)	51(14.3)	
Agriculture, Laborer, Unemployed, Other	17(4.9)	36(10.0)	
**Smoking status**			
Nonsmoker	305(93.3)	318(89.1)	0.066
Ex-smoker	12(3.7)	28(7.8)	
Current smoker	10(3.0)	11(3.1)	
**Alcohol consumption (g/day)**			
0	175(56.6)	190(53.1)	0.144
0< ≡ 14.9	119(38.5)	137(38.2)	
> 15	15(4.9)	31(8.7)	
**Physical activity^b ^(Met-min/week)**			
≤ 396	77(22.9)	59(16.6)	0.076
396-<1272	91(27.0)	114(32.0)	
1272-<2772	81(24.0)	101(28.4)	
≥ 2772	88(26.1)	82(23.0)	
**Age at menarche (years)**			
≤ 13	97(28.9)	91(25.4)	< 0.001
14	74(22.0)	97(27.1)	
15	86(25.6)	53(14.8)	
≥ 16	79(23.5)	117(32.7)	
**Menopausal status**			
No	196(54.4)	210(58.7)	0.254
Yes	164(45.6)	148(41.3)	
**Age at menopause^c ^(years)**			
< 46	37(26.6)	43(29.5)	0.697
46-<49	35(25.2)	31(21.2)	
49-<52	39(28.1)	47(32.2)	
≥ 52	28(20.1)	25(17.1)	
**Type of menopause^c^**			
Natural	109(75.2)	98(66.7)	0.109
Surgery, Other	36(21.8)	49(33.3)	
**Postmenopausal hormone use^c^**			
Never	88(62.0)	115(80.4)	< 0.001
Ever	54(38.0)	28(19.6)	
**Parity**			
No	46(12.8)	31(8.7)	0.074
Yes	314(87.2)	327(91.3)	

n (%) or mean ± SD

^a ^mean ± SD, ^b ^Metabolic equivalent units (METs) are multiples of the resting metabolic rate and calculated using the short form (version 2.0, April 2004) of the International Physical Activity Questionnaire (IPAQ), ^c ^postmenopausal women.

**Table 2:** Comparison of food and energy intake of the study subjects

	Total			Premenopausal women			Postmenopausal women		
	
	Control	Case	*P*	Control	Case	*p*	Control	Case	*P*
	(n = 360)	(n = 358)		(n = 196)	(n = 210)		(n = 164)	(n = 148)	
Total fish (g/day)	24.1 ± 21.1/17.5	21.8 ± 21.3/15.5	0.012	22.9 ± 18.1/18.2	21.7 ± 21.1/14.8	0.214	25.6 ± 24.1/17.1	22.0 ± 21.8/16.2	0.022
Lean fish	12.0 ± 11.2/8.5	14.1 ± 14.9/9.2	0.916	10.6 ± 8.5/8.3	13.4 ± 14.1/8.5	0.494	13.6 ± 13.6/9.4	15.1 ± 16.1/10.5	0.637
Fatty fish	12.1 ± 13.4/8.1	7.6 ± 9.7/4.5	< 0.001	12.3 ± 13.1/8.7	8.2 ± 10.3/5.0	< 0.001	11.9 ± 13.9/6.3	6.8 ± 8.8/3.6	< 0.001
ω-3 fatty acid (g/day)	0.228 ± 0.278/0.143	0.168 ± 0.227/0.090	< 0.001	0.216 ± 0.294/0.128	0.179 ± 0.244/0.098	0.089	0.242 ± 0.259/0.157	0.152 ± 0.201/0.079	< 0.001
EPA(20:5n-3)	0.085 ± 0.147/0.041	0.054 ± 0.089/0.025	< 0.001	0.083 ± 0.172/0.035	0.057 ± 0.098/0.027	0.017	0.089 ± 0.111/0.044	0.050 ± 0.075/0.022	< 0.001
DHA(22:6n-3)	0.174 ± 0.261/0.092	0.115 ± 0.174/0.056	< 0.001	0.166 ± 0.296/0.082	0.123 ± 0.192/0.061	0.084	0.184 ± 0.213/0.105	0.104 ± 0.146/0.051	< 0.001
Energy (kcal/day)	1752.5 ± 548.5	1813.8 ± 492.9	0.032	1797.6 ± 574.9	1811.1 ± 460.8	0.353	1698.5 ± 511.6	1817.8 ± 536.8	0.039

Data are presented as mean ± standard deviation/median.

EPA: Eicosapentaenoic acid, DHA: Docosahexaenoic acid

Table 3 shows the risk of breast cancer in relation to fish intake in both age-adjusted and multivariate-adjusted models. After adjusting for confounding variables in the multivariate logistic regression models, there was a protective effect of fatty fish intake for all study subjects in the highest quartile (OR [95% CI], *p *for trend: 0.23 [0.13 to 0.42], *p *< 0.001) compared to the lowest. The protective effect of fatty fish intake was observed in both pre- and postmenopausal women.

**Table 3:** Odds ratios of breast cancer risk according to level of fish intake

	Total				Premenopausal women					Postmenopausal women		
	
	Control (n)	Case (n)	Age adjusted Odds ratio	Multivariate Odds ratio^a^	Control (n)	Case (n)	Age adjusted Odds ratio	Multivariate Odds ratio^b^	Control (n)	Case (n)	Age adjusted Odds ratio	Multivariate Odds ratio^c^
Total fish (g/day)												
< 9.99	90	122	1.00 (referent)	1.00 (referent)	47	70	1.00 (referent)	1.00 (referent)	43	52	1.00 (referent)	1.00 (referent)
9.99-<17.51	90	80	0.65(0.43–0.98)	0.64(0.38–1.07)	49	52	0.69(0.40–1.19)	0.57(0.27–1.19)	41	28	0.57(0.30–1.08)	0.55(0.26–1.19)
17.51-<33.70	90	80	0.65(0.43–0.98)	0.57(0.34–0.95)	56	44	0.52(0.30–0.90)	0.38(0.18–0.78)	34	36	0.89(0.48–1.67)	1.02(0.47–2.22)
≥ 33.70	90	76	0.62(0.41–0.93)	0.55(0.32–0.96)	44	44	0.65(0.37–1.14)	0.49(0.22–1.10)	46	32	0.59(0.32–1.09)	0.62(0.28–1.39)
P for trend			0.054	0.063			0.157	0.094			0.205	0.475

Lean fish (g/day)												
< 4.63	90	98	1.00 (referent)	1.00 (referent)	52	56	1.00 (referent)	1.00 (referent)	38	42	1.00 (referent)	1.00 (referent)
4.63-<8.53	90	70	0.71(0.46–1.08)	0.74(0.43–1.26)	49	45	0.84(0.48–1.46)	0.86(0.42–1.78)	41	25	0.53(0.27–1.04)	0.43(0.19–0.98)
8.53-<15.27	89	70	0.72(0.47–1.10)	0.61(0.36–1.04)	52	45	0.78(0.45–1.36)	0.60(0.29–1.22)	37	25	0.62(0.31–1.22)	0.50(0.22–1.16)
≥ 15.27	91	120	1.20(0.81–1.79)	1.21(0.72–2.04)	43	64	1.34(0.78–2.32)	1.22(0.58–2.57)	48	56	1.07(0.59–1.92)	1.02(0.47–2.21)
P for trend			0.102	0.236			0.181	0.551			0.286	0.328

Fatty fish (g/day)												
< 3.42	90	147	1.00 (referent)	1.00 (referent)	44	63	1.00 (referent)	1.00 (referent)	44	69	1.00 (referent)	1.00 (referent)
3.42-<8.18	90	79	0.53(0.36–0.80)	0.65(0.39–1.08)	50	69	0.64(0.37–1.16)	0.65(0.31–1.35)	44	30	0.43(0.24–0.79)	0.64(0.31–1.31)
8.18-<15.39	90	91	0.61(0.41–0.91)	0.54(0.32–0.90)	52	55	0.62(0.37–1.04)	0.50(0.25–0.99)	34	32	0.61(0.33–1.14)	0.64(0.29–1.42)
≥ 15.39	90	41	0.27(0.17–0.44)	0.23(0.13–0.42)	50	23	0.29(0.16–0.54)	0.19(0.08–0.45)	42	17	0.26(0.13–0.52)	0.27(0.11–0.66)
P for trend			**< .001**	**< .001**			**< 0.001**	**< 0.001**			**< 0.001**	**0.005**

^a ^adjusted for age, BMI, family history of breast cancer, supplement use, education level, occupation, alcohol consumption, smoking status, physical activity, parity, total energy intake, menopausal status, age at menarche; ^b ^adjusted for age, BMI, family history of breast cancer, supplement use, education level, occupation, alcohol consumption, smoking status, physical activity, parity, total energy intake, age at menarche; ^c ^adjusted for age, BMI, family history of breast cancer, supplement use, education level, occupation, alcohol consumption, smoking status, physical activity, parity, total energy intake, postmenopausal hormone use, age at menarche. Energy-adjusted nutrient intakes were computed as the residuals from the regression model with total caloric intake as the independent variable and absolute nutrient intake as the dependent variable.

Table 4 presents the odds ratios of breast cancer risk with regard to ω-3 fatty acid intake. Among premenopausal women, there was a significant reduction in breast cancer risk for the highest intake quartiles of ω-3 fatty acids (ORs [95% CI]: 0.46 [0.22 to 0.96], compared to the reference group who consumed the lowest quartile of intake. However, there was no significant association between EPA or DHA intake and breast cancer risk in premenopausal women. After adjusting for confounding variables in the multivariate logistic regression models, postmenopausal subjects consuming more than 0.101 g of EPA and 0.213 g of DHA from fish per day showed a 62% and 68% decreased breast cancer risk compared to the reference group (who consumed less than 0.014 g of EPA and 0.037 g of DHA per day), respectively. In contrast, there was no statistically significant difference in any quartile category compared to the lowest intake of ω-3 fatty acids, although p for trend was marginally significant (p = 0.068).

**Table 4:** Odds ratios of breast cancer risk according to ω-3 fatty acid intake level

	Total				Premenopausal women				Postmenopausal women			
	
	Control (n)	Case (n)	Age adjusted Odds ratio	Multivariate Odds ratio^a^	Control (n)	Case (n)	Age adjusted Odds ratio	Multivariate Odds ratio^b^	Control (n)	Case (n)	Age adjusted Odds ratio	Multivariate Odds ratio^c^
ω-3 fatty acid (g/day)												
< 0.059	90	134	1.00 (referent)	1.00 (referent)	47	73	1.00 (referent)	1.00 (referent)	43	61	1.00 (referent)	1.00 (referent)
0.059-<0.143	90	90	0.67(0.45–1.00)	0.83(0.50–1.37)	57	55	0.61(0.36–1.03)	0.83(0.42–1.64)	33	35	0.76(0.41–1.41)	1.01(0.48–2.15)
0.143-<0.296	90	76	0.56(0.37–0.85)	0.74(0.44–1.24)	46	46	0.62(0.36–1.08)	0.83(0.40–1.70)	44	30	0.49(0.26–0.90)	0.76(0.35–1.61)
≥ 0.296	90	58	0.43(0.28–0.66)	0.47(0.27–0.80)	46	36	0.49(0.28–0.87)	0.46(0.22–0.96)	44	22	0.35(0.18–0.68)	0.51(0.22–1.13)
P for trend			< 0.001	**0.004**			0.037	**0.040**			0.001	**0.068**

EPA(20:5n-3) (g/day)												
< 0.014	90	124	1.00 (referent)	1.00 (referent)	54	66	1.00 (referent)	1.00 (referent)	36	58	1.00 (referent)	1.00 (referent)
0.014-<0.041	95	103	0.79(0.53–1.17)	0.90(0.55–1.48)	53	63	0.96(0.57–1.61)	1.11(0.57–2.15)	42	40	0.61(0.33–1.12)	0.81(0.38–1.73)
0.041-<0.101	85	76	0.65(0.43–0.98)	0.91(0.54–1.55)	47	45	0.78(0.45–1.35)	1.13(0.54–2.33)	38	31	0.52(0.27–0.98)	0.78(0.35–1.74)
≥ 0.101	90	55	0.44(0.28–0.68)	0.50(0.28–0.91)	42	36	0.68(0.38–1.22)	0.67(0.30–1.50)	48	19	0.25(0.12–0.49)	0.38(0.15–0.96)
P for trend			< 0.001	**0.016**			0.182	0.227			< 0.001	**0.035**

DHA(22:6n-3) (g/day)												
< 0.037	90	132	1.00 (referent)	1.00 (referent)	52	72	1.00 (referent)	1.00 (referent)	38	60	1.00 (referent)	1.00 (referent)
0.037-<0.092	90	90	0.68(0.46–1.01)	0.86(0.52–1.43)	52	54	0.74(0.44–1.26)	0.91(0.46–1.78)	38	36	0.61(0.33–1.14)	0.90(0.42–1.95)
0.092-<0.213	90	86	0.65(0.43–0.97)	0.77(0.46–1.28)	48	49	0.72(0.42–1.23)	0.93(0.46–1.85)	42	37	0.56(0.31–1.04)	0.81(0.37–1.75)
≥ 0.213	90	50	0.37(0.24–0.58)	0.44(0.24–0.79)	44	35	0.56(0.32–1.00)	0.54(0.24–1.20)	46	15	0.21(0.10–0.42)	0.32(0.13–0.82)
P for trend			< 0.001	**0.004**			0.075	0.118			< 0.001	**0.010**

^a ^adjusted for age, BMI, family history of breast cancer, supplement use, education level, occupation, alcohol consumption, smoking status, physical activity, parity, total energy intake, menopausal status, age at menarche; ^b ^adjusted for age, BMI, family history of breast cancer, supplement use, education level, occupation, alcohol consumption, smoking status, physical activity, parity, total energy intake, age at menarche; ^c ^adjusted for age, BMI, family history of breast cancer, supplement use, education level, occupation, alcohol consumption, smoking status, physical activity, parity, total energy intake, postmenopausal hormone use, age at menarche. EPA: Eicosapentaenoic acid, DHA: Docosahexaenoic acid. Energy-adjusted nutrient intakes were computed as the residuals from the regression model with total caloric intake as the independent variable and absolute nutrient intake as the dependent variable.

## Discussion

The results of studies investigating the association between ω-3 fatty acids and breast cancer risk vary according to the study design. A meta-analysis of biomarker studies based on three cohort and seven case-control studies found a significant protective effect for total ω-3 PUFAs, but only an inverse association with borderline significance for α-linolenic acid in case-control studies. The authors suggested that the findings of cohort studies fit well with the hypotheses of experimental animal studies [9]. However, according to a recent systematic review, one study showed a significantly increased risk for breast cancer, three studies showed a decreased risk, and seven studies failed to show a significant association with ω-3 fatty acids intake [20]. A study of women from New York City found no apparent association between fish intake and breast cancer risk [12,14,21]. Consistent with this, a large-scale EPIC study [10], and studies conducted in Norway [22] and Sweden [23] found no apparent evidence for an association between fish intake and breast cancer risk. Holmes *et al*. reported a 9% increase in risk with a 0.1% increase in energy from ω-3 fatty acids in the Nurses' Health Study [24].

In addition to study design, ethnic groups have also responded differently in these studies. For instance, a Japanese population demonstrated a significant decrease in postmenopausal breast cancer risk with increased fish intake [25], and breast cancer risk was inversely associated with erythrocyte compositions of EPA (OR, 0.27; 95% CI, 0.14–0.53 for the highest to the lowest tertile; p for trend < 0.001), DHA (OR, 0.06; 95% CI, 0.02–0.16; p for trend < 0.001), and ω-3 PUFAs (OR, 0.11; 95% CI, 0.05–0.24; p for trend < 0.001) as biomarkers [26]. A similar trend was found in another Japanese study performed by Wakai et al[13], which detected a significant decrease in breast cancer risk in the highest quartile of fish fat and long-chain ω-3 fatty acids intake compared with the lowest; the relative risks were 0.56 (95% CI: 0.33–0.94) and 0.50 (95% CI: 0.30–0.85), respectively. The Singapore Chinese Healthy Study demonstrated that high levels of dietary ω-3 fatty acids from fish/shellfish were significantly associated with a reduced risk for breast cancer [11]. Compared to the lowest quartile of intake, individuals in the top three quartiles exhibited a 26% reduction in risk. In ecological studies in the Netherlands [7] and Canada [8], there was an increase in consumption of fish and fish ω-3 PUFAs that may contribute to a lower breast cancer risk. A study of Norwegian women found an inverse relationship between breast cancer risk and consumption of poached fish, although there was no association with overall fish intake [27]. Additionally, in the UK, fish oil consumption has been associated with protection against breast carcinogenesis [28,29]. A postmenopausal study conducted in the US found a significant inverse association between fish intake (canned, fried, fresh, and shellfish) and breast cancer risk [30].

Many factors may contribute to these discrepant findings in various regions, including sample size, adjustment for potentially confounding variables, the detail and quality of the dietary assessment, unmeasured changes in diet over time, and the stage of cancer at diagnosis [31]. Alternatively, the study discrepancies could also be explained by other two possibilities, either differences in the range of fish intake or interactions between ω-3 fatty acids and antioxidant components in the diet [32]. For example, fish consumption in Japan and Korea is much higher than in the United States [33]. The mean daily consumption of 24.1 g of total fish identified by this study, consists of 2.3% of total daily energy intake, but the US population consumed only 0.74% of their total energy from fish [33]. The proportion was 6.21% in the Japanese population [33]. It is also possible that low variability in fish or ω-3 fatty acids intake in each individual or non-differential misclassification of estimated ω-3 fatty acid intake played a role in these results [34]. Alternatively, findings from animal studies have suggested that the strength of the association with marine ω-3 fatty acids may be reduced in the presence of high antioxidant intake, which has been proposed to inhibit the formation of lipid peroxidation products [35,36]. There are still more possible reasons for these inconsistencies. Halogenated hydrocarbons, including polychlorinated biphenyls and dichlorodiphynyltrichloroethane, or heavy metals that are concentrated in fish may exert estrogenic effects that could predispose women to breast cancer [16,37]. In addition, genetic backgrounds, such as polymorphisms in glutathione S-transferase, may modify the effect of marine ω-3 fatty acids [38]. We also can not exclude the possibility that inconsistent results between epidemiological studies are due to measurement errors associated with dietary assessment, as these are inherent in a retrospective study design [19]. However, it remains possible that other nutrients or micronutrients in fish are partly responsible for the inverse association [39,40].

A study of metastatic mouse mammary carcinoma demonstrated that a diet containing α-linolenic acid-rich linseed oil was very effective in arresting tumor progression in mice [4]. In addition, tumor growth and metastases formation were inhibited by diets including fish oil [5] or EPA or DHA [6]. Larsson proposed several molecular mechanisms for the potential effect of ω-3 PUFAs on carcinogenesis: 1) suppression of arachidonic acid-derived eicosanoid biosynthesis, 2) influence on transcription factor activity, gene expression, and signal transduction, 3) alteration of estrogen metabolism, 4) increased and decreased production of free radicals and reactive oxygen species, and 5) effect on insulin sensitivity and membrane fluidity [34]. For example, EPA and DHA cause a concentration-dependent inhibition of breast cancer cell growth [41,42]. Another possible mechanism could involve inhibition of cyclooxygenase and p21 gene expression and up-regulation of p53 gene expression [43,44].

The present study demonstrated that there were significantly different effects of ω-3 fatty acids from fish on breast cancer risk in pre-and postmenopausal women. Reasons for the stronger associations in postmenopausal women are not yet clear. With respect to the etiologies of pre- and postmenopausal breast cancer, several hypotheses are possible [45,46]. The relationship between dietary fat intake and breast cancer risk in premenopausal women may differ from that in postmenopausal women. Adiposity and reproductive factors act reversely on the sensitivity of breast cancer tissue [46,47]. One study found that postmenopausal patients had significantly lower levels of DHA in breast adipose tissue compared to controls with benign breast disease [48]. It is also plausible that diet has a stronger impact on breast cancer risk during early adult life than later in life [49]. Maillard et al. [50] and Bagga et al. [51] confirmed that long-chain ω-3 fatty acids have a beneficial effect in postmenopausal women, using breast adipose tissue as a biomarker.

The present study is the first to explore the relationship between fish and fish ω-3 fatty acid intake with breast cancer risk in a Korean population. The data were gathered in a detailed face-to-face interview, which enabled the collection of comprehensive information on related lifestyle factors, thus lessening the potential for misclassification and measurement errors. In spite of such strengths, this study also possesses some of the limitations usually inherent to case-control study designs (i.e., selection and recall biases). In particular, the control group was more likely to be highly educated or a professional/office worker, which suggests that participants enrolled from the cancer screening program may over-represent those with healthier habits as opposed to their community-based counterparts. Well-known menstrual risk factors for breast cancer, such as early age at menarche, late age at menopause, or hormone replacement therapy use, did not show definitive associations in the current study population. However, high body mass index and other hormone-related risk factors showed a positive association with breast cancer risk. Cancer patients may differ from controls in their recall of dietary habits. For this reason, the interviewer tried to collect information as soon as possible after diagnosis, which was typically right after surgery. In addition, a wide range of potentially confounding factors, including demographic and lifestyle characteristics, still need to be considered. We were also constrained by our inability to identify other sources of dietary ω-3 fatty acids. The addition of supplements may have enabled us to identify the impact of total ω-3 fatty acids intake. Notably, this study did not include information on fish species (cod, salmon, mullet, etc.), preparation methods (frying, deep frying, poaching, etc.), how long the fish was cooked, or how the fish was consumed (with sauce, vegetable, salted, etc.). These factors may help to elucidate the mechanism whereby fish intake is associated with decreased breast cancer risk. Moreover, further investigations into the dietary intake of halogenated hydrocarbons or heavy metals and genetic factors will be important in clarifying the preventive effect of fish intake on breast cancer.

## Conclusion

This investigation has identified fish and fish ω-3 fatty acid intake as an important potential protective factor in the nutritional etiology of breast cancer. Our results revealed an inverse relation between breast cancer risk and dietary intake of fatty fish and ω-3 fatty acids from fish. These findings will provide the basis for further studies.

## Competing interests

The authors declare that they have no competing interests.

## Authors' contributions

JK conceived of the study, participated in its design and coordination, and drafted the manuscript. S-YL participated in the coordination of the study and performed the statistical analysis. AS participated in the coordination of the study and helped to draft the manuscript. JR and E-SL participated in the design of the study and revising the manuscript critically for important intellectual content. All authors read and approved the final manuscript.

## Pre-publication history

The pre-publication history for this paper can be accessed here:

http://www.biomedcentral.com/1471-2407/9/216/prepub
